# Feasibility of using a patient portal to recruit Latino adults into a smoking cessation and physical activity randomized clinical trial: A secondary analysis

**DOI:** 10.18332/tid/219277

**Published:** 2026-07-26

**Authors:** Victoria Chavez Uceda, Rafael H. Orfin, Estefania Panizoni, Julia LanzDuret-Hernandez, Alejandra Hurtado-deMendoza, Arlette Chávez-Iñiguez, Daniel Fuller, David X. Marquez, Deborah J. Ossip, Dongmei Li, Elizabeth Vasquez, Jeffrey W. Ramos-Santiago, Lisa Carter Bawa, Rachel M. Aleese, Reza Yousefi Nooraie, Scott McIntosh, Ana Paula Cupertino, Francisco Cartujano-Barrera

**Affiliations:** 1 Department of Public Health Sciences, University of Rochester Medical Center, New York, United States; 2 Georgetown Lombardi Comprehensive Cancer Center, Georgetown University, Washington, District of Columbia, United States; 3 Community Health and Epidemiology, University of Saskatchewan, Saskatoon, Canada; 4 Department of Kinesiology and Nutrition, University of Illinois Chicago, Chicago, United States; 5 Clinical and Translational Science Institute, University of Rochester Medical Center, New York, United States; 6 Department of Epidemiology and Biostatistics, University at Albany, New York, United States; 7 Department of Surgery, University of Rochester Medical Center, New York, United States; 8 Center for Discovery and Innovation, Hackensack Meridian Health, New Jersey, United States; 9 Information Systems Division, University of Rochester Medical Center, New York, United States

**Keywords:** patient portal, electronic health record, smoking cessation, physical activity, Latino adults

## Abstract

**INTRODUCTION:**

Emerging efforts are using patient portals to send recruitment messages to potential participants in randomized clinical trials (RCTs). We assessed the feasibility of using a patient portal to recruit Latino adults into a smoking cessation and physical activity RCT.

**METHODS:**

Secondary analysis of an ongoing RCT. In 2025, the electronic health record (EHR) at the University of Rochester Medical Center (URMC) was utilized to identify patients with the following characteristics: Hispanic/Latino ethnicity, aged ≥18 years, currently smoking cigarettes, and seen at URMC within the last five years. Identified patients with an active account on MyChart^®^, the patient portal at URMC, were messaged up to two times. The study team made up to five phone call attempts to patients who expressed interest in further explaining the study and completing eligibility screening. Feasibility was defined as exceeding a 1% overall enrollment rate (number of patients who enrolled divided by the number of patients invited).

**RESULTS:**

Two thousand patients were identified in the EHR at URMC as potentially eligible. A total of 1122 patients had an active MyChart account and were messaged (56.1%, 1122/2000). One hundred thirty patients viewed the message (11.6%, 130/1122) and 53 responded as interested in the study (4.7%, 53/1122). The study team successfully enrolled thirteen patients, resulting in an overall enrollment rate of 1.2% (13/1122).

**CONCLUSIONS:**

It is feasible to use a patient portal to recruit Latino adults into a smoking cessation and physical activity RCT. While modest, our overall enrollment rate provides important evidence to inform future recruitment efforts, particularly given its high reach and low implementation effort.

## INTRODUCTION

Patient portals play a central role in modern healthcare by providing patients with secure, digital access to their electronic health records (EHR)^1^. However, disparities in access and use exist across racial and ethnic groups^1^. Latino adults report lower rates of patient portal access (72.2%) compared with Black (79.9%) and White (88.1%) adults^1^.

Emerging efforts have demonstrated the feasibility of using patient portals to send recruitment messages to potential participants in randomized clinical trials (RCTs)^2-4^. However, little is known about their feasibility for recruiting Latino patients, who have historically been underrepresented in smoking cessation RCTs^5^. Given low access to and use of patient portals among Latinos^1^, assessing feasibility is critical to determine whether this recruitment effort can reach the Latino community. If feasible, patient portals may offer an important avenue for improving the representation of Latinos in RCTs and for addressing health disparities. The purpose of this study was to assess the feasibility of using a patient portal to recruit Latino adults into a smoking cessation and physical activity RCT.

## METHODS

This study is a secondary analysis of the recruitment conducted through a patient portal for ‘Actívatexto’, an ongoing RCT (ClinicalTrials.gov identifier: NCT06926608) assessing the efficacy of a mobile intervention that promotes smoking cessation and physical activity among Latino adults^6^. Study procedures for ‘Actívatexto’ were approved by the University of Rochester Medical Center (URMC) Institutional Review Board (protocol number STUDY00009837).

In September 2025, the EHR at URMC was utilized to identify patients with the following characteristics: Hispanic/Latino ethnicity, aged ≥18 years, currently smoking cigarettes, and seen at URMC within the last five years. Between September and October 2025, identified patients with an active account on MyChart^®^, the patient portal at URMC, were messaged up to two times, in accordance with institutional policies. An active MyChart account was defined as one in which the patient created a username and password and successfully logged into the patient portal at least once. The MyChart message, sent in both English and Spanish, said that a remote study to help Latinos quit smoking and become more physically active was offering supportive text messages, a Fitbit^®^ device, and nicotine patches or gum at no cost, with up to $90 for participating.

The study team made up to five phone call attempts to patients who expressed interest in further explaining the study and completing eligibility screening. Voicemails were left when patients did not answer the call, requesting that they return the call to the study team. Eligible patients self-identified as Hispanic and/or Latino, were able to communicate in English and/or Spanish, were aged .18 years, did not meet the recommended levels of physical activity [at least 150 minutes of moderate to vigorous of moderate to vigorous activity (MVPA) per week]^7^, smoked cigarettes at least 3 days per week^8,9^, were interested in quitting smoking in the next 30 days, had a cellphone with text messaging capability, did not share a cellphone device with someone else, knew how to send and read text messages, were willing to wear a wearable device to monitor physical activity on a daily basis, and were willing to complete three study visits. Exclusion criteria were: use of tobacco products other than cigarettes in the past 30 days (including e-cigarettes), being pregnant, breastfeeding, or planning to become pregnant in the next year, planning to move from their current residence in the following six months, another household member participating in the study, and being unable to become more physically active or engage in MVPA^10^. Eligible patients were scheduled for a Zoom^®^ or telephone session, during which the study team reviewed the study and obtained informed consent. All study procedures were available in English and Spanish.

Feasibility was defined as exceeding a 1% overall enrollment rate, consistent with prior studies^3,4^. The overall enrollment rate was defined as the number of patients who enrolled divided by the number of patients invited. Navar et al.^3^ analyzed data from 23 studies that used MyChart messages for recruitment and included 84062 patients, 9.5% of whom were Latino. The overall enrollment rate across these studies was 1.2%^3^. Chávez-Iñiguez et al.^4^ analyzed data from a single study using MyChart messages with 668 patients, all Latino, and reported an overall enrollment rate of 1.3%^4^.

Viewing and overall study interest rates were also assessed to provide additional context on recruitment performance. Viewing rate was defined as the number of patients who viewed the recruitment message divided by the number of patients invited. The overall study interest rate was defined as the number of patients who expressed interest in participating in the study divided by the number of patients invited.

## RESULTS

**Figure 1:**
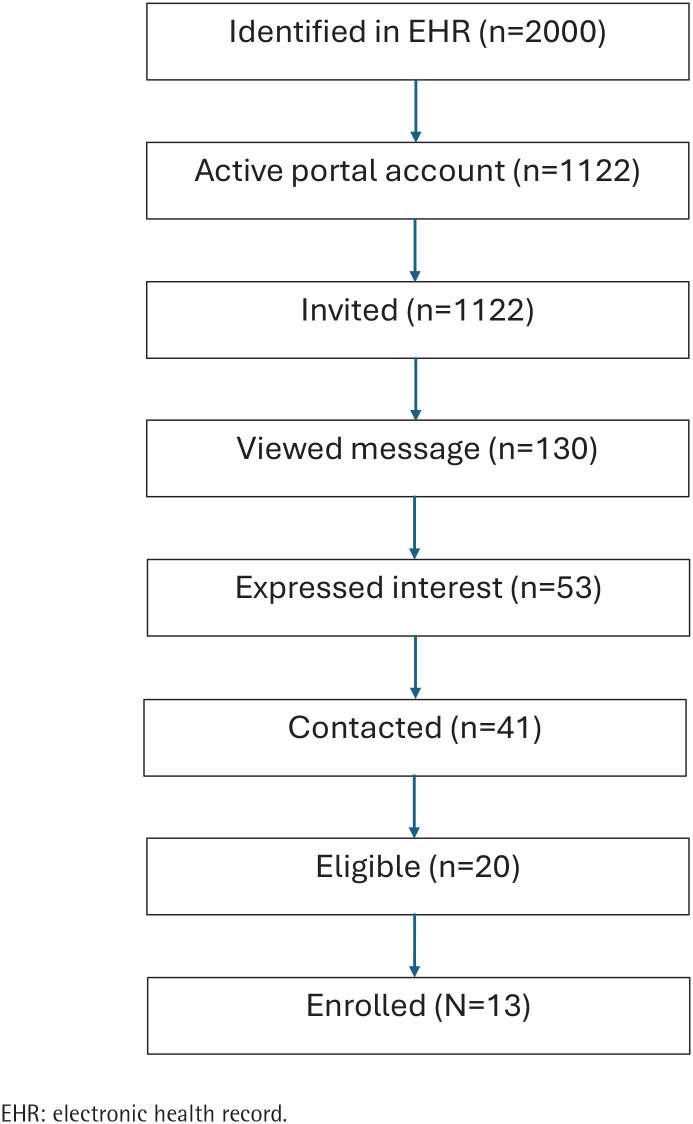
Flow diagram of using a patient portal to recruit Latino adults into a smoking cessation and physical activity randomized clinical trial, September-October 2025

Two thousand patients were identified in the EHR at URMC as potentially eligible (Figure 1). A total of 1122 patients had an active MyChart account and were invited to participate in the study through a MyChart message (56.1%, 1122/2000). One hundred thirty patients viewed the message, and 53 responded as interested in the study. The viewing and overall study interest rates were 11.6% (130/1122) and 4.7% (53/1122), respectively.

Of the 53 patients who expressed interest, 12 could not be contacted (22.6%, 12/53), and 41 were successfully contacted by the study team (77.4%, 41/53). Of the 41 patients who were contacted by the study team, 20 were eligible (48.8%, 20/41), 17 were ineligible (41.5%, 17/41), and 4 were no longer interested (9.7%, 4/41). Reasons for ineligibility included not being able to become more physically active or engage in MVPA (e.g. experiencing dizziness or chest pain, using a walker; 47.1%, 8/17), use of tobacco products other than cigarettes (i.e. e-cigarettes; 41.2%, 7/17), no longer smoking (23.5%, 4/17), meeting the recommended levels of physical activity (11.8%, 2/17), not self-identifying as Hispanic and/or Latino (5.9%, 1/17), and planning to move from their current residence in the following six months (5.9%, 1/17).

The study team successfully enrolled thirteen patients, resulting in an overall enrollment rate of 1.2% (13/1122).

## DISCUSSION

To our knowledge, this is the first study to assess the feasibility of using a patient portal to recruit Latino adults into a smoking cessation and physical activity RCT. Consistent with previous research^2,3^, Latino patients in this study had lower rates of active MyChart accounts (56.1%) compared with the broader patient population at URMC (67%)^2^. Nonetheless, the patient portal was deemed feasible, as the observed overall enrollment rate (1.2%) exceeded the established benchmark (1%). While modest, this rate provides important evidence to inform future recruitment efforts, particularly given its high reach and low implementation effort. Moreover, this study strengthens existing evidence and adds to the expanding literature indicating that Latino adults are interested in participating in smoking cessation research^11-13^.

In our study, each patient received up to two messages. The viewing rate (11.6% vs. 34.8%) and overall study interest rate (4.7% vs 7.4%) were substantially lower than those reported by Navar et al.^3^. However, the overall enrollment rate was comparable. The lower viewing and interest rates may reflect differences in message frequency or other unmeasured factors, highlighting the need for further research to determine whether higher viewing rates could translate into greater study interest and enrollment. This is important because previous studies have demonstrated that increasing the number of invitations via text messages enhances enrollment rates among Latino adults in smoking cessation interventions^14^.

In our study, vaping was the single leading reason that interested patients did not meet eligibility criteria. This is noteworthy, as vaping has not emerged as a major exclusion factor in previous smoking cessation studies with Latino adults^8^. However, this pattern aligns with national data indicating that vaping is increasing within the Latino community^15^, alongside initial research efforts on vaping cessation among Latino young adults^16^. These results underscore the impact of vaping-related eligibility criteria on study enrollment in smoking cessation RCTs.

### Limitations

Some limitations should be noted when interpreting these findings. First, the study was conducted within a single health system (URMC), and outcomes may not generalize to other settings. Second, messages sent to patients were limited to providing study information, and the second message was identical to the first one. Future research could examine whether modifying message content (e.g. messages highlighting the benefits of quitting smoking) or using sequential messages with distinct wording (e.g. the second message highlights a lack of response) might influence study interest and enrollment rates. Third, up to five phone call attempts were made to patients who expressed interest to explain the study and complete eligibility screening. It is possible that making more attempts may yield different enrollment outcomes. Fourth, the messages mentioned multiple incentives for participating in the RCT (e.g. free nicotine replacement therapy, financial compensation). This may have influenced study interest and overall enrollment rates. Finally, the current data do not allow us to examine whether the characteristics of patients who did not view or respond to the message, or enroll in the RCT, differed from those who did.

## CONCLUSIONS

It is feasible to use a patient portal to recruit Latino adults into a smoking cessation and physical activity RCT. While modest, our overall enrollment rate provides important evidence to inform future recruitment efforts, particularly given its high reach and low implementation effort.

## Data Availability

The data supporting this research are available from the authors on reasonable request.
